# PD-1+ T-Cells Correlate with Nerve Fiber Density as a Prognostic Biomarker in Patients with Resected Perihilar Cholangiocarcinoma

**DOI:** 10.3390/cancers14092190

**Published:** 2022-04-27

**Authors:** Xiuxiang Tan, Jan Bednarsch, Mika Rosin, Simone Appinger, Dong Liu, Georg Wiltberger, Juan Garcia Vallejo, Sven Arke Lang, Zoltan Czigany, Shiva Boroojerdi, Nadine T. Gaisa, Peter Boor, Roman David Bülow, Judith De Vos-Geelen, Liselot Valkenburg-van Iersel, Marian C. Clahsen-van Groningen, Evelien J. M. de Jong, Bas Groot Koerkamp, Michail Doukas, Flavio G. Rocha, Tom Luedde, Uwe Klinge, Shivan Sivakumar, Ulf Peter Neumann, Lara Rosaline Heij

**Affiliations:** 1 Department of Surgery and Transplantation, University Hospital RWTH Aachen, 52074 Aachen, Germany; xtan@ukaachen.de (X.T.); jbednarsch@ukaachen.de (J.B.); mika.rosin@rwth-aachen.de (M.R.); simone.appinger@rwth-aachen.de (S.A.); dliu@ukaachen.de (D.L.); gwiltberger@ukaachen.de (G.W.); svlang@ukaachen.de (S.A.L.); zczigany@ukaachen.de (Z.C.); shiva.boroojerdi@rwth-aachen.de (S.B.); uklinge@ukaachen.de (U.K.); uneumann@ukaachen.de (U.P.N.); 2 NUTRIM School of Nutrition and Translational Research in Metabolism, Maastricht University, 6229 HX Maastricht, The Netherlands; 3 Department of Molecular Cell Biology and Immunology, VU University Medical Center, 1081 HV Amsterdam, The Netherlands; jj.garciavallejo@amsterdamumc.nl; 4 Institute of Pathology, University Hospital RWTH Aachen, 52074 Aachen, Germany; ngaisa@ukaachen.de (N.T.G.); pboor@ukaachen.de (P.B.); rbuelow@ukaachen.de (R.D.B.); 5 Department of Internal Medicine, Division of Medical Oncology, GROW School for Oncology and Developmental Biology, Maastricht University Medical Centre, 6200 MD Maastricht, The Netherlands; judith.de.vos@mumc.nl (J.D.V.-G.); liselot.van.iersel@mumc.nl (L.V.-v.I.); evelien.de.jong@mumc.nl (E.J.M.d.J.); 6 Department of Pathology, Erasmus University Medical Center, 3015 GD Rotterdam, The Netherlands; m.clahsen-vangroningen@erasmusmc.nl (M.C.C.-v.G.); m.doukas@erasmusmc.nl (M.D.); 7 Department of Surgery, Erasmus MC Cancer Institute, 3015 GD Rotterdam, The Netherlands; b.grootkoerkamp@erasmusmc.nl; 8 Division of Surgical Oncology, Knight Cancer Institute, Oregon Health and Science University, Portland, OR 97239, USA; rochaf@ohsu.edu; 9 Department of Gastroenterology, Hepatology and Infectious Diseases, University Hospital Duesseldorf, 40225 Düsseldorf, Germany; tom.luedde@med.uni-duesseldorf.de; 10 Kennedy Institute of Rheumatology, University of Oxford, Oxford OX3 7FY, UK; shivan.sivakumar@oncology.ox.ac.uk; 11 Department of Oncology, University of Oxford, Oxford OX3 7DQ, UK; 12 Department of Surgery, Maastricht University Medical Centre (MUMC), 6229 HX Maastricht, The Netherlands

**Keywords:** cholangiocarcinoma, liver cancer, tumor microenvironment, nerve fiber density, immune checkpoint, immune cells

## Abstract

**Simple Summary:**

Recent studies have identified Nerve Fiber Density (NFD) as a prognostic biomarker for Cholangiocarcinoma (CCA). In the field of CCA treatment with checkpoint inhibitors (ICI) is increasing but not all patients respond. Good biomarkers to predict response to ICI are lacking. The present study investigates the immune cell composition and expression of checkpoint molecules in relation to NFD in perihilar cholangiocarcinoma (pCCA) patients. Our study identified NFD to correlate with PD-1+ T cells as a biomarker indicative for a good prognosis.

**Abstract:**

Background and Aims: Perihilar cholangiocarcinoma (pCCA) is a hepatobiliary malignancy, with a dismal prognosis. Nerve fiber density (NFD)—a novel prognostic biomarker—describes the density of small nerve fibers without cancer invasion and is categorized into high numbers and low numbers of small nerve fibers (high vs low NFD). NFD is different than perineural invasion (PNI), defined as nerve fiber trunks invaded by cancer cells. Here, we aim to explore differences in immune cell populations and survival between high and low NFD patients. Approach and Results: We applied multiplex immunofluorescence (mIF) on 47 pCCA patients and investigated immune cell composition in the tumor microenvironment (TME) of high and low NFD. Group comparison and oncological outcome analysis was performed. CD8+PD-1 expression was higher in the high NFD than in the low NFD group (12.24 × 10^−6^ vs. 1.38 × 10^−6^ positive cells by overall cell count, *p* = 0.017). High CD8+PD-1 expression was further identified as an independent predictor of overall (OS; Hazard ratio (HR) = 0.41; *p* = 0.031) and recurrence-free survival (RFS; HR = 0.40; *p* = 0.039). Correspondingly, the median OS was 83 months (95% confidence interval (CI): 18–48) in patients with high CD8+PD-1+ expression compared to 19 months (95% CI: 5–93) in patients with low CD8+PD-1+ expression (*p* = 0.018 log rank). Furthermore, RFS was significantly lower in patients with low CD8+PD-1+ expression (14 months (95% CI: 6–22)) compared to patients with high CD8+PD-1+ expression (83 months (95% CI: 17–149), *p* = 0.018 log rank). Conclusions: PD-1+ T-cells correlate with high NFD as a prognostic biomarker and predict good survival; the biological pathway needs to be investigated.

## 1. Introduction

Cholangiocarcinoma (CCA) is a rare and aggressive hepatobiliary malignancy arising from the biliary tract. Based on the cancer location within the biliary tree, CCA is classified into three subtypes: intrahepatic cholangiocarcinoma (iCCA), perihilar cholangiocarcinoma (pCCA) and distal cholangiocarcinoma (dCCA). CCA usually has a five-year overall survival (OS) of less than 10% [1]. Surgical resection remains the only curative treatment for these patients, however, only a minority of patients is eligible for surgery as CCA is often diagnosed at an advanced stage and resection is no longer an option. For almost all CCA patients, conventional cytotoxic chemotherapy is the mainstay treatment option [2], resulting in a survival benefit of only months in comparison to best supportive care and causing toxic side-effects. The upcoming treatment options within personalized medicine have not brought much for the group of patients with pCCA [3]. Recent trials have opened the field of immunotherapy as a treatment option displaying the possibility of long-term survival in some patients [4]. For CCA patients this is a developing field and results from phase 3 trials are expected [5,6,7]. Based on phase 1 clinical trials there is hope that immunotherapy in combination with chemotherapy regimens might improve outcomes in CCA patients as well [8].

The low success rate of CCA treatment is caused by many factors, and limited knowledge of its tumor microenvironment (TME) contributes to this problem. CCA has a high heterogeneity at the genomic, epigenetic and molecular level, hence, primary CCA contains a diverse range of cell types [9,10,11,12,13,14]. Furthermore, the TME is a host for many different immune cells and stimulatory and inhibitory effects take place. PCCA shows abundant desmoplastic stroma, which contains many immune cells, providing either a host protective immune environment or facilitating tumor progression [15]. Immune cell compositions play an important role in the immune response to the cancer and different phenotypes have been suggested in combined hepatocellular-cholangiocarcinoma patients (cHCC-CCA) [16] and in iCCA [17].

From a histopathological point of view, pCCA characteristically has an extensive stromal component, in which complex microenvironment interactions take place [15]. In the past decade, great efforts have been made to explore the complexity of the TME and to develop novel therapies that might help to improve oncological outcomes. However, more needs to be discovered about the spatial relationship among cells within the complex TME and their expression patterns of co-stimulatory and inhibitory signals to understand the response to immune checkpoint blockades in clinical trials. However, not every patient responds equally and response rates differ from <5% to >40% depending on the cancer type. There is an urgent need for biomarkers to better predict responses to immunotherapy [18]. Predictors for an anti-tumor response to ICIs currently are: high PD-L1 expression; microsatellite instable cancers or microsatellite high (MSI-H) cancers; tumor infiltrating lymphocytes (TILs) at the edge of the tumor and high mutational burden (TMB). Unfortunately, even in the presence of one of these markers, not all patients seem to respond to ICIs [14,19,20,21,22]. Furthermore, we are in urgent need of new biomarkers to predict responses to checkpoint inhibitors to facilitate patient selection.

**Figure 1:**
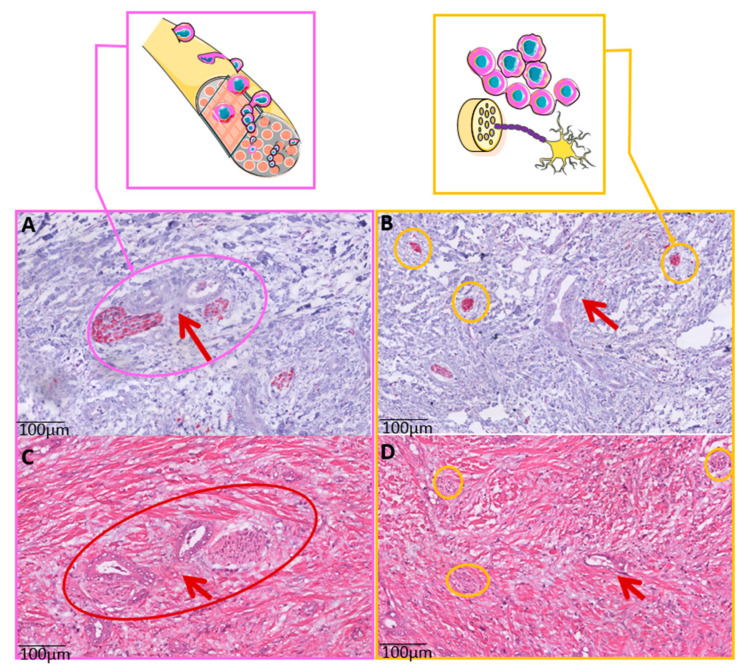
The difference between Perineural Invasion (PNI) and Nerve Fiber Density (NFD). (**A**) PNI is defined as tumor cells invading the perineurium of the nerve. In the neuronal marker (PGP9.5) staining, the nerve fiber in red (red arrow) surrounded and invaded by tumor cells and glandular structures. (**B**) NFD shows the presence of small nerve fibers in the tumor microenvironment (TME). These nerve fibers are small in diameter (<100 μm) and do not show any invasion of tumor cells. The red arrow points to the tumor cells and the yellow circles mark the presence of small nerve fibers stained with the neuronal marker (PGP9.5). (**C**) Corresponding H&E staining of PNI. PNI is recognizable for the pathologist. (**D**) On the H&E, the cancer is recognizable, however the small nerve fibers are not detectable on this routine staining. H&E, hematoxylin and eosin; NDF, nerve fiber density; PNI, perineural invasion; TME, tumor microenvironment.

We have recently shown that nerve fiber density (NFD) in the TME functions is an important prognostic biomarker in CCA patients. NFD is associated with clinical outcomes in pCCA and iCCA patients [23] and patients with the presence of small nerve fibers in the TME display a better survival. The underlying mechanisms of this clinical observation are not discovered yet. Of vital importance is the difference of a well-known aggressive feature known as perineural invasion (PNI), which shows the invasion of cancer cells into the nerve fibers (Figure 1). This histological feature is detectable on a routine H&E staining and it is thought that the perineurium of the nerve fiber is a barrier for the chemotherapy to reach the cancer. Moreover, the nerve fiber environment provides a way of least resistance for the tumor to spread and progress. NFD has an opposite effect on outcome and consists of nerve fibers growing in the TME. In case of high NFD, small nerve fibers grow into the TME. These nerve fibers are only visualized by a special staining and the nerve fibers are smaller in diameter and usually do not show any tumor invasion.

Given this prognostic value of NFD in pCCA, we hypothesized NFD might also be associated with different immunophenotypes and therefore used multiplex immunofluorescence (mIF) to reveal the differences in immune cell composition and distribution combined with the expression of co-stimulatory and co-inhibitory checkpoint markers.

## 2. Materials and Methods

### 2.1. Patient Cohort

In total, 47 pCCA formalin fixed paraffin embedded (FFPE) tissue blocks were selected from the archive of the University Hospital RWTH Aachen. All patients were treated and operated on in our hospital between 2010 and 2019. Of the 47 patients, two individuals were excluded due to poor quality of the slide after staining, resulting in 45 patients being eligible for this analysis. The study was conducted in accordance with the requirements of the Institutional Review Board of the RWTH-Aachen University (EK 106/18), the Declaration of Helsinki, and good clinical practice guidelines (ICH-GCP).

### 2.2. Surgical Techniques, Adjuvant Treatment and Follow-Up

All patients who were referred for surgical treatment of CCA to our institution underwent a detailed clinical work-up, which included an oncological staging in accordance with common standards and radical surgery with lymphadenectomy as previously described [23,24,25] Patients treated between 2010–2017 were recommended for adjuvant therapy in case of positive lymph nodes or an R1 resection. After 2017 every patient was recommended for adjuvant therapy in line with the BILCAP trial [26]. The postoperative follow up consisted of clinical follow up at the local hospital or with an oncologist with laboratory testing (CA19-9) and imaging. Confirmation of recurrence was performed by histology or radiology.

### 2.3. Whole Slide Immunohistochemistry (IHC) and Nerve Fiber Counts

All samples were checked for the presence of tumor region by hematoxylin and eosin-stained sections. Slides were cut in tissue sections (2.5 μm thick) from formalin-fixed blocks, deparaffinized in xylene and rehydrated in graded alcohols. Slides were boiled in citrate buffer (pH 6.0) at 95–100 °C for 5 min and were cooled for 20 min at room temperature with endogenous peroxide in methanol for 10 min. Then, these slides were incubated with rabbit anti-human PGP 9.5 (DAKO 1:100) overnight at 4 °C to mark the nerve fibers. Histological Slides were scanned using the whole-slide scanners Aperio AT2 with ×40 objective (Leica Biosystems, Wetzlar, Germany), corresponding to a pixel-edge-length of = 0.252. A single digital image per case was uploaded in Qupath 0.1.6.

NFD nerve fiber counts from our previous study were used for immune cell phenotyping [24]. The NFD method was evaluated by manually counting the number of nerve fascicles with diameters of <100 μm in 20 continuous visual fields at ×200 magnification [27]. Based on NFD results, patients were categorized into a low NFD group (<10 nerve fibers) and a high NFD group (≥10 nerve fibers) as previously described (24).

### 2.4. Whole Slide Multiplex Immunofluorescence (mIF)

All FFPE samples were subjected to multiplex immunofluorescence (mIF) in serial 5.0 μm histological tumor sections obtained from representative FFPE tumor blocks. The FFPE blocks were carefully selected within the presence of the tumor region. The sections were labeled by using the Opal 7-Color fIHC Kit (PerkinElmer, Waltham, MA, USA). The antibody fluorophores were grouped into a panel of five antibodies. The order of antibodies staining was always kept constant on all sections and sections were firstly counterstained with DAPI (Vector Laboratories, Eching, Germany). The multiplex immunofluorescence panel consisted of CD8, CD68, PD-1, PD-L1, and PD-L2 (Table 1). All antibodies were diluted with Antibody Diluent (with Background Reducing Components, Dako, Germany). Secondary antibodies were applied with ImmPRESS™ HRP (Peroxidase) Polymer Detection Kit (Vector Laboratories, Burlingame, CA, USA). TSA reagents were diluted with 1×  Plus Amplification Diluent (PerkinElmer/Akoya Biosciences, Marlborough, MA 01752, USA).

The manual for mIF is described as Edwin R. Parra’s protocol [28]: in short, the first marker was incubated after the FFPE sections were deparaffinized in xylene and rehydrated in graded alcohols. The second marker was applied on the following day, and the third marker was applied on the third day. After all five sequential reactions, sections were finally cover-slipped with VECTRASHIELD^®^ HardSet™ Antifade Mounting Medium.

The slides were then digitally scanned with the TissueFAXS PLUS system (TissueGnostics, Vienna, Austria). Image analysis was performed in two regions of interest (ROI) in each image (only if present in the slide: tumor region and tumor free region). The size of the ROI varied per slide. Immune cell expression was calculated in percentages throughout the whole project.

**Figure 2:**
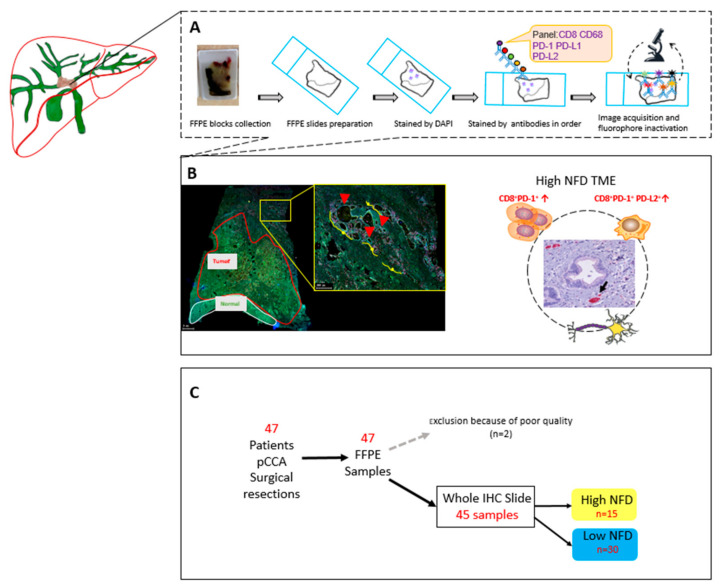
Overview of study workflow. (**A**) The formalin-fixed paraffin-embedded (FFPE) blocks were collected from the pathology archives. The slides were cut and stained with DAPI and five antibodies. The immunofluorescence-stained slides were scanned. (**B**) The digital scans were annotated for different regions: Tumor and Tumor-free. Cells were subsequently counted in these separate regions. For the NFD patients, the slides were selected based on the small nerve fiber count from previous work and the cell counting was conducted in the tumor region. (**C**) In total, we included 47 patients in this study. From 45 patients we were able to analyze the digital scans. DAPI, diamidino-2-phenylindole, FFPE; formalin-fixed paraffin-embedded.

Strataquest software was used to analyze the antibody staining and cell counts. The library information was used to associate each fluorochrome component with a mIF marker. All immune cell populations were quantified as positive cells divided by the overall cell count using the cell segmentation, and thresholds were set manually under supervision of two pathologists (LH/MC). Positive cell count was categorized based on thresholds, and a value above the threshold was considered as positive. Checks were performed by the pathologists (LH/MC). See Figure 2 for an overview of workflow. 

### 2.5. Statistical Analysis

Clinical variables and immune cell data and the difference between patients with high and low NFD were investigated by Mann–Whitney U test for continuous variables and the χ^2^ test or Fisher’s exact test in accordance with scale and number count. Furthermore, the overall survival (OS) and the recurrence-free survival (RFS) of the cohort were determined by the Kaplan–Meier method. The OS was defined as the date of surgery to the date of death based on any cause, while the RFS was defined as the date of surgery to the date of first tumor recurrence. Associations between the OS or RFS and clinical or multiplex variables were determined by univariate and multivariate Cox Regression analyses. Survival curves were generated by the Kaplan–Meier method and compared with the log-rank test. The cut-off level for group categorization for survival analysis was determined by the receiver operating characteristic (ROC)-analysis of the OS with respect to the analyzed continued variable as previously described [24]. The level of significance was set to α = 0.05, and *p* values were calculated using two-sided testing.

## 3. Results

### 3.1. Patients’ Characteristics

The study cohort comprised of 45 individuals with 15 patients in the high NFD and 30 patients in the low NFD group. Demographical features, e.g., gender (*p* = 0.664), age (*p* = 0.563) and American College of Anesthesiologists (ASA) status (*p* = 0.850) displayed no difference between the groups. Furthermore, no statistical differences were observed with respect to basic pathological features as T category (*p* = 0.324), N category (*p* = 0.832), vascular invasion (*p* = 0.225), lymphatic invasion (*p* = 0.611), perineural invasion (*p* = 0.773) and tumor grading (*p* = 0.085). More details on clinicopathological features are displayed in Table 2.

### 3.2. Multiplex Data

The 45 slides all included whole slide analysis for the combined for immune cell markers (CD8 and CD68) and immune checkpoint markers (PD-1, PD-L1 and PD-L2). The corresponding H&E slide of the same block was used to annotate the tumor region and positive cells were counted. DAPI nuclei staining was used to generate a total cell count. We assessed differences in immune cell counts (CD8 and CD68) and expressions of checkpoint markers (PD-1, PD-L1 and PD-L2). Interestingly, the CD8+ and CD68+ numbers were not significantly different, although the PD-1 expressions were. We note that PD-L1 was not significantly expressed and this marker is used for patient selection for pembroluzimab.

**Figure 3:**
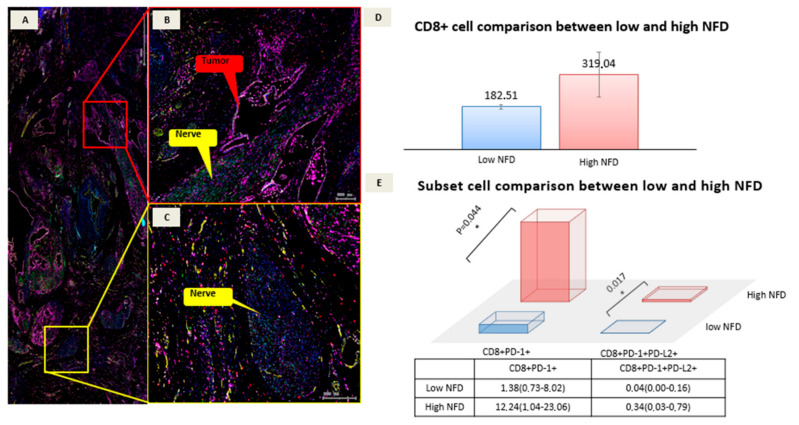
Multiplex immunofluorescence (mIF) digitized images. (**A**) Zoomed-in image of a slide with perihilar cholangiocarcinoma, with perihilar presence of many big nerve fibers and large vessels. (**B**) The red box visualizes PNI with the tumor glands highlighted with red and the nerve fiber marked in yellow. For PNI the tumor glands need to be orientated really close to the nerve and invade the perineurium. (**C**) The yellow box visualizes a large nerve fiber without tumor invasion. The increase in small nerve fibers, which are counted to assess NFD are not detectable without a neuronal marker. The positive cell counting was conducted in the tumor annotation in a patient with high NFD. (**D**) For high and low NFD, positive cell counts for CD8 were not significantly different the tumor region. (**E**) Cell subset comparison. CD8+PD-1+ (*p* = 0.044) and CD8+PD-1PD-L2 cell counts (*p* = 0.017) were significantly higher in patients with high NFD. NFD, nerve fiber density; PNI, perineural invasion.

While the expression of CD8+ and CD68+, as well as the expression of co-stimulatory signals, appear to be tangentially higher in the high NFD cohort, a pronounced statistical effect was overserved for CD8+PD-1+ and CD8+PD-1+PD-L2+ cells. CD8+PD-1 expression was higher in the high NFD than in the low NFD group (12.24 × 10^−6^ vs. 1.38 × 10^−6^, *p* = 0.017) as was CD8+PD-1+PD-L2+ (0.34 × 10^−6^ vs. 0.04 × 10^−6^, *p* = 0.044; Table 2). See Figure 3 for the CD8 cell comparison between low vs high NFD. 

### 3.3. Survival Analysis

As high expression of CD8+PD-1+ and CD8+PD-1+PD-L2+ were associated with high NFD, these two variables were further included into a survival analysis for the whole cohort. For this purpose, a ROC analysis evaluating CD8+PD-1+ and CD8+PD-1+PD-L2+ expression with respect to OS was carried out and cut-off values for these variables determined with respect to the optimized accuracy and equal weight for sensitivity and specificity errors. By this approach, the cut-off values were determined to be <1.4 × 10^−6^ (= low expression) vs. ≥1.4 × 10^−6^ (= high expression) for CD8+PD-1+ and <0.1 × 10^−6^ (= low expression) vs. ≥0.1 × 10^−6^ (= high expression) for CD8+PD-1+PD-L2+.

**Figure 4:**
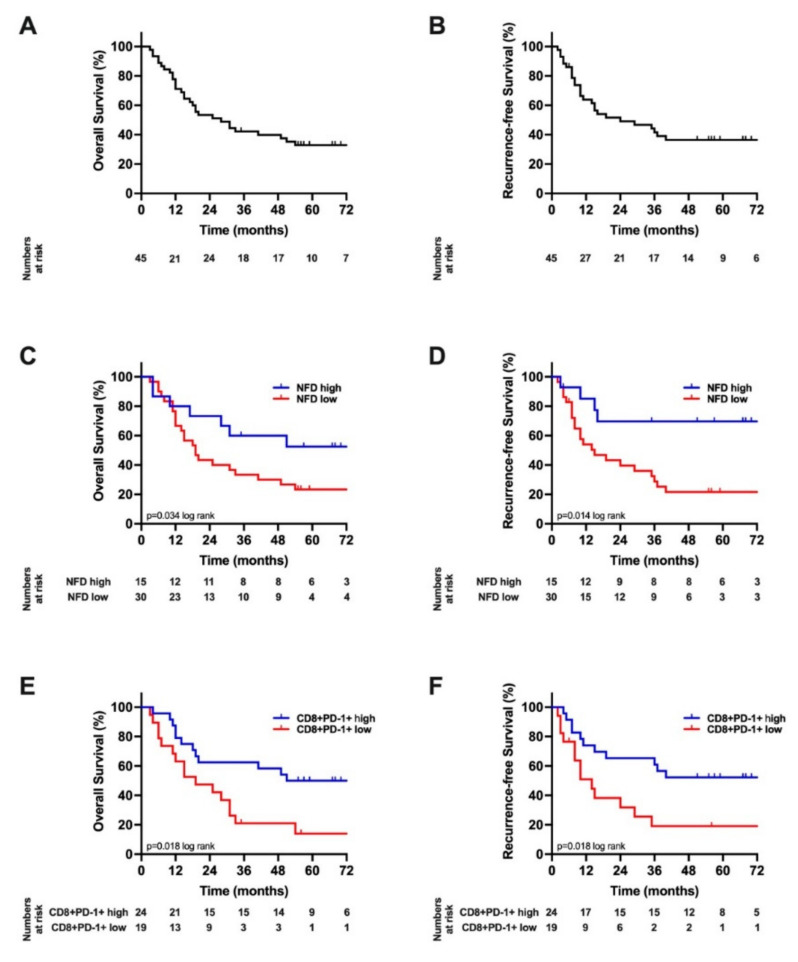
Oncological survival in CCA with respect to CD8+PD1+ count and nerve fiber density: (**A**) Overall survival. The median OS of the cohort was 28 months; (**B**) Recurrence-free survival. The median RFS of the cohort was 24 months; (**C**) Overall survival stratified by nerve fiber density. The median OS of the cohort was 19 months in patients with low NFD and 90 months in patients with high NFD; (**D**) Recurrence-free survival stratified by nerve fiber density. The median RFS of the cohort was 15 months in patients with low NFD and 83 months in patients with high NFD; (**E**) Overall survival stratified by CD8+PD1+ count. The median OS of the cohort was 19 months in patients with low CD8+PD1+ expression and 83 months in patients with high CD8+PD1+ ≥expression; (**F**) Recurrence-free survival stratified by CD8+PD1+ count. The median RFS of the cohort was 14 months in patients with low CD8+PD1+ expression and 83 months in patients with high CD8+PD1+ expression. NFD, nerve fiber density; OS, overall survival; RFS, recurrence-free survival.

After a median follow-up of 70 months, the median OS of the cohort was 28 months (95% Confidence interval (CI): 12–43) and the RFS 24 months (95% CI: 0–49; Figure 4A,B). A Kaplan–Meier analysis with respect to NFD showed a median OS of 90 months (95% CI: 0–196) in patients with high NFD compared to 19 months (95% CI: 12–27) in patients with low NFD (*p* = 0.037 log rank, Figure 4C). Furthermore, the RFS was significantly lower in patients with low NFD (15 months (95% CI: 3–37)) compared to patients with high NFD (70 months (95% CI: 48 –93), *p* = 0.014 log rank, Figure 4D).

A similar survival analysis was conducted for CD8+PD-1+ expression. Here, a Kaplan–Meier analysis showed a median OS of 83 months (95% CI: 18–48) in patients with high CD8+PD-1+ expression compared to 19 months (95% CI: 5–93) in patients with low CD8+PD-1+ expression (*p* = 0.018 log rank, Figure 4E). Furthermore, RFS was significantly lower in patients with low CD8+PD-1+ expression (14 months (95% CI: 6–22)) compared to patients with high CD8+PD-1+ expression (83 months (95% CI: 17–149), *p* = 0.018 log rank, Figure 4F).

### 3.4. Cox Regression Analysis

To further explore independent prognostic markers of survival in our cohort, Cox regression analyses were conducted. Here, in univariate analysis, tumor grading (Hazard ratio (HR) = 3.22; *p* = 0.010) and high CD8+PD-1+ expression (HR = 0.44; *p* = 0.029) were significantly associated with OS. All variables showing *p* value <0.10 were included in a multivariable Cox regression model. Here, tumor grading (HR = 3.67; *p* = 0.010) and high CD8+PD-1+ expression (HR = 0.42; *p* = 0.031) were identified as independent predictors for improved OS (Table 3).

In an analog univariate analysis, high NFD (HR = 0.31; *p* = 0.021), tumor grading (HR = 4.79; *p* = 0.001) and high CD8+PD-1+ expression (HR = 0.54; *p* = 0.024) showed significant associations with RFS. In the corresponding multivariable Cox regression model, tumor grading (HR = 5.51; *p* = 0.001) and high CD8+PD-1+ expression (HR = 0.40; *p* = 0.039) were identified as independent predictors of RFS (Table 4).

## 4. Discussion

PCCA is considered a rare primary biliary tract cancer and therefore it remains understudied. While the literature is short of large cohorts of patients, reported outcomes are entirely poor compared to other solid malignancies, especially for those individuals who are not eligible for a surgical resection.

Immunotherapy remains experimental in the clinical treatment of pCCA and patient stratification for systemic treatment, especially in the context of immunotherapy, is the subject of ongoing investigation. For HCC, as the most common primary liver cancer, immunotherapy in combination with Bevacizumab is already a first line treatment in the palliative setting [29]. First clinical trial results report that pCCA is an immune responsive malignancy indicating a potential role of immunotherapy in improving patients’ survival. However, only a subset of patients might respond to immunotherapy and biomarkers to identify these individuals for immunotherapy are needed urgently [30].

The histology of pCCA usually shows characteristic growth pattern of nerves invaded by cancer cells. Even though most patients have this feature somewhere present in the tumor, not all patients display poor outcomes. In this study, we have shown that patients with high NFD is associated with a higher PD-1 expression. These patients do further display significant better oncological outcome survival. The underlying mechanism for this still needs to be further investigated.

NFD is defined as large numbers of small nerve fibers in the TME, these nerve fibers are not invaded by cancer cells. A recent study has demonstrated that CD8+ infiltration was associated with better survival in patients with iCCA [31]. Hence, we evaluated the clinical significance of the main immune cells (T cells and macrophages) in pCCA patients. In our previous publication [24], we investigated the role of NFD in a large pCCA cohort and demonstrated high NFD being independently associated with improved survival after surgical resection. We subsequently hypothesized that the small nerve fibers attract immune cells providing a better immune response to the cancer. Patients with a high NFD have abundant CD8+PD-1+ T-cells.

This finding identifies a subgroup of pCCA patients with a better survival. Further, these might suggest this subgroup for immunotherapy-based adjuvant treatment.

PD-1 checkpoint therapy unleashes the immune cells blocked by PD-1 expanding the T cell population at the interface and in the tumor. Potentially the high NFD subgroup of patients could benefit from immunotherapy, when the CD8+ T-cells blocked with PD-1 are reactivated. Our data are in line with previous findings on HCC patients, where patients with high levels of PD-1 expression showed an improved survival [32] and low counts of CD8 T-cells were indicative of a poorer outcome [33]. Previous work on the immune landscape in intrahepatic cholangiocarcinoma showed an immunosuppressive environment with low numbers of CD3 and CD4 to be correlated with reduced long-term outcomes [34] and low expression of PD-1 to be associated with an improved oncological survival [35,36,37]. For extrahepatic cholangiocarcinoma, high numbers of CD3+ T-cells combined with expression of PD-L1 on the tumor cells correlated with a more invasive growth [38]. The prognostic relevance of the PD-1 marker is therefore diverse, however expression of this checkpoint receptor usually indicates patients are likely to benefit from immunotherapy [39,40,41]. Previous work has shown that cholangiocarcinoma patients with high densities of tumor infiltrating lymphocytes also have high expression levels of PD-L1 [42]. Besides using the expression of PD-1 and PD-L1 to predict outcome, different immune responses in biliary tract cancer are indicative for a better or worse outcome. The presence of tumor infiltrating lymphocytes are also an important prognostic factor [43]. The different prognostic values of the PD-1 and PD-L1 expression in cholangiocarcinoma patients suggest that expression of this marker by itself is not enough to function as a good biomarker.

The TME is host to many different cell types and nerve fibers seem to play a dual role. The large nerve fiber trunks with tumor invasion are usually a sign of a bad outcome [44] and the small nerve fibers are indicative of a good outcome [23]. The same phenomenon exists for immune cells (Tregs are usually bad for prognosis and CD8 cytoxic T cells are protective to the host [45]) and fibroblasts [46]. In light of this perspective, we hypothesized that nerve fibers have a dual role as well and they potentially can be used to stratify patients for response to immunotherapy.

In the future our findings need to be validated by external cohorts and underlying pathways should be identified. Once the pathways behind high NFD are known, the next step would be to see if high NFD can be influenced by therapeutics. First this needs to be conducted in 3D models and hopefully later, potentially, a nerve fiber targeted therapy could be included in a clinical trial and maybe nerve fiber targeted therapy can be part of a combination (chemo)therapy.

We have started a functional study to validate our findings in 3D models and hopefully this will be the next step to a clinical implementation.

Our study has limitations. Unfortunately, our cohort of only 50 surgically resected perihilar patients makes the sample size limited. The study could be validated in a larger sample size, using an external validation cohort, and this would strengthen the study. To collect larger cohorts, international collaboration is needed. Furthermore, our method could be improved by using a multiplex imaging device with the accessibility of a 40-antibody panel, like CODEX or Hyperion. Of course, these methods are expensive.

## 5. Conclusions

To our knowledge, this is the first study in pCCA using a wide multiplex antibody panel focusing on immune cells in the TME in combination with checkpoint markers in relation to NFD. PD-1 expression correlates with high NFD patients suggesting NFD can be used as a simple prognostic biomarker. NFD can be easily integrated in the routine workup of the pathology report, since only one neuronal antibody is needed to achieve a nerve fiber count.

## Figures and Tables

**Table 1:** Monoclonal antibodies in the multiplex immunofluorescence panel.

Antibody	Marker	Dilution	Incubation	Theme	Manufacturer
CD8	Cytotoxic T	1:500	30 min	CySO	Dako
CD68	Macrophage	1:6000	30 min	Cy7E	Dako
PD-1/CD279	Checkpoint	1:250	Over night	Cy47	Abcam
PD-L1/CD274	Checkpoint	1:200	Over night	46HE	Dako
PD-L2/CD273	Checkpoint	1:400	Over night	43HE	Abcam

**Table 2:** Comparative analysis of surgically treated patients with respect to nerve fiber density.

Variables	NFD Group		
	High (n = 15)	Low (n = 30)	*p* Value
Demographics			
Gender, m/f (%)	10 (66.7)/5 (33.3)	18 (60)/12 (40)	0.664
Age (years)	70 (58–72)	64 (55–73)	0.563
ASA, n (%)			0.850
I	1 (6.7)	1 (3.3)	
II	8 (53.3)	15 (50.0)	
III	6 (40.0)	13 (43.3)	
IV	0	1 (3.3)	
**Pathological examination**			
T category, n (%)			0.324
T1	0	0	
T2	8 (53.4)	20 (66.6)	
T3	6 (40.0)	6 (20.0)	
T4	1 (6.7)	4 (13.3)	
N category			0.832
N0	7 (46.7)	13 (43.6)	
N1	8 (53.3)	17 (56.7)	
Vascular invasion, n (%)	2 (13.3)	10 (33.3)	0.225
Lymphatic invasion, n (%)	5 (33.3)	7 (23.3)	0.611
Perineural invasion, n (%)	10 (66.7)	21 (87.5)	0.733
Tumor grading, n (%)			0.085
G1	0	0	
G2	14 (93.3)	20 (71.4)	
G3	0	7 (25.0)	
G4	0	1 (3.6)	
**Multiplex Imaging Data**			
CD8-Panel (×10^−6^)			
CD8+	319.04 (131.37–448.66)	182.51 (118.95–347.54)	0.195
CD8+PD-1+	12.24 (1.04–23.06)	1.38 (0.73–8.02)	**0.017**
CD8+PD-1+PD-L1+	0.90 (0.03–1.97)	0.27 (0.04–0.66)	0.228
CD8+PD-1+PD-L1+PD-L2+	0.14 (0.00–0.52)	0.02 (0.00–0.08)	0.150
CD8+PD-1+PD-L2+	0.34 (0.03–0.79)	0.04 (0.00–0.16)	**0.044**
CD8+PD-L1+	6.53 (1.92–15.76)	2.83 (1.16–10.42)	0.306
CD8+PD-L1+PD-L2+	0.58 (0.16–5.41)	0.13 (0.05–0.80)	0.091
CD8+PD-L2+	3.30 (0.81–17.37)	2.36 (0.37–4.93)	0.097
CD68-Panel (×10^−6^)			
CD68+	709.33 (348.70–1475.20)	505.81 (282.94–692.45)	0.087
CD68+PD-1+	5.82 (1.22–15.16)	2.52 (0.85–5.53)	0.140
CD68+PD-1+PD-L1+	1.01 (0.06–1.90)	0.37 (0.05–1.09)	0.363
CD68+PD-1+PD-L1+PLD2+	0.01 (0.00–0.47)	0.02 (0.00–0.14)	0.946
CD68+PD-1+PD-L2+	0.11 (0.02–1.39)	0.04 (0.00–0.53)	0.513
CD68+PD-L1+	15.31 (1.58–42.57)	6.64 (3.41–13.88)	0.195
CD68+PD-L1+PD-L2+	1.18 (0.08–4.36)	0.06 (0.34–0.97)	0.120
CD68+PD-L2+	10.24 (4.03–24.87)	4.93 (2.56–8.84)	0.070
**Follow-up Data**			
Recurrence-free survival (months)	70 (48–93)	15 (3–27)	**0.014**
Overall survival (months)	90 (0–196)	19 (12–27)	**0.037**

Data presented as median and interquartile range if not noted otherwise. Multiplex data is presented as positive cells per overall cell count of the tumor ROI. Follow-up data is presented as median and 95% CI. Categorical data were compared using the chi-squared test, fisher’s exact test or linear-by-linear association according to scale and number of cases. Data derived from continuous variables of different groups were compared by Mann–Whitney-U-Test. Follow-up data was calculated by the Kaplan–Meier-Method and compared by log rank tests. ASA, American society of anesthesiologists’ classification; CI, confidence interval. ROI, region of interest.

**Table 3:** Uni- and multivariate analysis of overall survival.

Variables	Univariate Analysis		Multivariate Analysis	
	HR (95% CI)	*p* Value	HR (95% CI)	*p* Value
NFD (low = 1)	0.47 (0.21–1.06)	0.070	excluded	0.595
Gender (male = 1)	1.22 (0.60–2.49)	0.576		
Age (<65 years = 1)	1.22 (0.61–2.46)	0.572		
ASA (I/II = 1)	1.14 (0.57–2.30)	0.707		
T category (T1/T2 = 1)	1.53 (0.76–3.09)	0.233		
N category (N0 = 1)	1.70 (0.83–3.49)	0.149		
Vascular invasion (No = 1)	1.75 (0.81–3.78)	0.154		
Lymphatic invasion (No = 1)	1.43 (0.65–3.15)	0.377		
Perineural invasion (No = 1)	1.50 (0.44–5.08)	0.515		
Tumor grading (G1/G2 = 1)	3.22 (1.33–7.82)	**0.010**	3.67 (1.37–9.82)	**0.010**
CD8+PD-1+ (low = 1)	0.44 (0.21–0.92)	**0.029**	0.42 (0.19–0.92)	**0.031**
CD8+PD-1+PD-L2+ (low = 1)	0.57 (0.26–1.22)	0.145		

Variables displaying a *p* value < 0.1 in the univariate Cox Regression were transferred into a multivariable Cox regression model.

**Table 4:** Uni- and multivariate analysis of recurrence-free survival.

Variables	Univariate Analysis		Multivariate Analysis	
	HR (95% CI)	*p* Value	HR (95% CI)	*p* Value
NFD (low = 1)	0.31 (0.12–0.84)	**0.021**	excluded	0.307
Gender (male = 1)	1.16 (0.54–2.49)	0.714		
Age (<65 years = 1)	1.03 (0.48–2.20)	0.941		
ASA (I/II = 1)	1.08 (0.51–2.31)	0.841		
T category (T1/T2 = 1)	1.24 (.57–2.73)	0.589		
N category (N0 = 1)	1.53 (0.71–3.31)	0.277		
Vascular invasion (No = 1)	2.24 (0.97–5.16)	0.059	excluded	0.085
Lymphatic invasion (No = 1)	1.46 (0.63–3.40)	0.377		
Perineural invasion (No = 1)	1.68 (0.39–7.28)	0.486		
Tumor grading (G1/G2 = 1)	4.79 (1.90–12.04)	**0.001**	5.51 (1.98–15.33)	**0.001**
CD8+PD-1+ (low = 1)	0.40 (0.18–0.89)	**0.024**	0.40 (0.17–0.96)	**0.039**
CD8+PD-1+PD-L2+ (low = 1)	0.54 (0.23–1.26)	0.156		

Variables displaying a *p* value < 0.1 in the univariate Cox Regression were transferred into a multivariable Cox regression model.

## Data Availability

Data will be made available upon request.

## Abbreviations

CCA	Cholangiocarcinoma
CHCC-CCA	Combined hepatocellular-cholangiocarcinoma
DCCA	Distal cholangiocarcinoma
FFPE	Formalin fixed paraffin embedded
HCC	Hepatocellular carcinoma
ICCA	Intrahepatic cholangiocarcinoma
MIF	Multiplex Immune Fluorescence
NFD	Nerve fib er density
OS	Overall survival
PCCA	Perihilar cholangiocarcinoma
PD-1	Programmed Death receptor
PD-L1	Programmed Death Ligand 1
PD-L2	Programmed Death Ligand 2
PGP 9.5	Protein gene-product 9.5
PNI	Perineural invasion
RFS	Recurrence-free survival
ROI	Region of interest
TME	Tumor microenvironment
